# Correlation of Serum Uric Acid With Cardiovascular Risk in Nigerian Patients

**DOI:** 10.7759/cureus.70285

**Published:** 2024-09-26

**Authors:** Ugochi C Okorafor, Chiamaka I Okorafor, Casmir Amadi, Okam Onyinye, Nnanna Achime

**Affiliations:** 1 Cardiology, Meridian Cardiac Center, Lagos, NGA; 2 Pharmacy, Festac Primary Health Center, Amuwo-Odofin Local Government Area, Lagos, NGA; 3 Internal Medicine/Cardiology, College of Medicine, University of Lagos, Lagos, NGA; 4 Internal Medicine, Alimosho General Hospital, Lagos, NGA

**Keywords:** hyperlipidemias, uric acid, risk score, cardiovascular risk factors, cardiovascular disease

## Abstract

Background: Studies exploring the relationship between serum uric acid (sUA) and cardiovascular risk in the Nigerian population remain sparse. The study aimed to assess the association between sUA levels and two measures of cardiovascular risk, namely the Framingham 10-year Cardiovascular Risk Score (FRS) and the Atherogenic Index of Plasma (AIP).

Methods: This retrospective study used data from clinical records of new, previously unregistered patients presenting at a private cardiac hospital over one year from November 2022 to October 2023. In total, 428 patients presented newly to the hospital in that period. The records of 138 patients were included in the project after various exclusions were made including for incomplete anthropometric and laboratory data. Statistical tests of association were used to determine the significance of the relationship between sUA levels and the measures of cardiovascular risk. Two-tailed p <0.05 was deemed statistically significant.

Results: Hyperuricemia was more prevalent in individuals with central obesity, i.e., waist circumference ≥94cm in males or 80cm in females (93.4% vs 6.6%; p=0.03). Serum uric acid also positively correlated with FRS (correlation coefficient: 0.190; p<0.05) and serum triglyceride levels and AIP (correlation coefficient: 0.259 and 0.294, respectively; p<0.001 for both). After multivariate analyses, uric acid was significantly and independently associated with high FRS and AIP after adjusting for age, smoking and diabetes history, blood pressure, total and high-density lipoprotein cholesterol, serum triglycerides and waist circumference (p<0.001).

Conclusion: The results emphasize the emergence of sUA as a pertinent cardiovascular risk factor in clinical settings. More research is needed to deduce the relationship, if any, between cardiovascular risk reduction and pharmacological reduction of sUA levels.

## Introduction

In various populations worldwide, elevated serum uric acid has been linked to cardiovascular disease (CVD) development and mortality [1]. A product of purine metabolism, uric acid, is a pro- or antioxidant depending on the chemical environment in vivo [2]. Although the cause of the association has not been clearly defined, high serum uric acid (sUA) is known to be closely related to cardiovascular risk factors such as hypertension, dyslipidaemia, impaired glucose metabolism and obesity and has been associated with increased severity of heart failure, atrial fibrillation and coronary artery disease [1,3,4]. Elevated uric acid also increases the risk of cardiovascular and all-cause mortality in patients with these conditions and is a marker for end-organ damage in hypertensive individuals [3,5-8]. However, it still has not been conclusively delineated whether uric acid is a causative factor, a risk factor or both. Assessment of the value of sUA as a possible risk factor is of clinical significance, particularly among patients attending a cardiology service, as these adults already have one or more cardiovascular risk factors. Early identification and modification of risk factors associated with cardiovascular disease development is paramount in managing these patients.

Studies detailing the association between sUA and cardiovascular risk scores in Nigerian patients are lacking. Hence, this study aimed to determine the prevalence of hyperuricemia amongst Nigerian patients in a clinical setting and investigate the presence, or lack thereof, of a relationship between sUA levels and two measures of cardiovascular risk, namely the Framingham 10-year Cardiovascular Risk Score (FRS) and the Atherogenic Index of Plasma (AIP). This research was previously presented as a meeting abstract at the 2024 Cardiology World Conference, Madrid, Spain, on September 7, 2024.

## Materials and methods

Study design

This retrospective study used the clinical records of patients presenting at a private cardiac facility in Lagos, Nigeria. The sample population consisted of patients presenting to the hospital's outpatient clinic for the first time between November 1, 2022, and October 31, 2023. The inclusion criteria used in this research were new patients, previously unregistered with the hospital, at least 18 years of age and who presented to the facility within the specified period (November 1, 2022 to October 31, 2023). The exclusion criteria were pregnant and newly postpartum women (waist circumference measurement could return false values), individuals for which the Framingham 10-year Cardiovascular Risk Score (FRS) could not be calculated (age <30 and >74 years and patients with a history of established cardiovascular disease, i.e. heart failure, peripheral arterial disease, myocardial infarction and stroke) and patients with missing anthropometric, clinical and laboratory data from their records. In total, 428 patients presented to the hospital within the specified period. After exclusions were made (222 had incomplete clinical, anthropometric or laboratory data and 68 were excluded because the FRS could not be calculated on the basis of age or presence of established cardiovascular disease), 138 clinical records remained eligible for inclusion in the study. Based on the study design, this research was granted a waiver by the Health Research Ethics Committee of the Lagos University Teaching Hospital, Idi-Araba, Lagos, Nigeria with identification number: ADM/DSCST/HREC/APP/6494.

Data collection

Anonymised and de-identified sociodemographic details, anthropometric measurements, medical histories and laboratory results were retrieved from the records into an electronic case report form. The variables were defined: serum uric acid above 7.0mg/dl in men and 6.0mg/dl in women was considered elevated [9]. Hypertension was defined as a history of previously diagnosed hypertension or patients currently on antihypertensives. Diabetes mellitus was diagnosed based on past medical history or those on treatment with antidiabetic medications. A positive smoking history was noted in patients who were current cigarette smokers.

Cutoffs for serum triglycerides and total, low and high-density lipoprotein cholesterol were obtained from the Adult Treatment Panel III document [10]. The cutoff for obesity was a body mass index (BMI) of at least 30kg/m^2^ [11]; central obesity was a waist circumference of at least 94cm in men and 80cm in women [12].

Cardiovascular risk assessment

The Framingham Risk Score was calculated to estimate each patient’s risk of developing cardiovascular disease in 10 years. The scores were categorised into low, moderate and high risk (< 10%, 10% to <20% and ≥20%, respectively) and computed using the age and sex of the patient, history of smoking, hypertension (and on antihypertensive medications) and diabetes, along with the values for systolic blood pressure, total and high-density lipoprotein (HDL) cholesterol [13]. Individuals with diabetes mellitus were also considered high risk regardless of previously computed scores. The Atherogenic Index of Plasma (AIP) is the logarithm of the ratio of serum triglycerides to HDL cholesterol. Scores less than 0.11 were considered low risk, 0.11-0.23 intermediate risk and ≥0.24 high risk [14].

Statistical analysis

The entire dataset was analysed using the Statistical Package for the Social Sciences (SPSS) software version 21 (SPSS Inc., Chicago, IL, USA). The data was summarised using descriptive statistics of frequency, percentage, mean and standard deviation. The Chi-squared test of association was used to determine the association between categorical variables, while analysis of variance (ANOVA) was employed when the analysis involved continuous variables. Linear regression analysis evaluated the association between the serum uric acid level, the FRS and the AIP. A confidence interval of 95% was used in the study, with p values <0.05 denoting statistical significance.

## Results

The sociodemographic, anthropometric and laboratory characteristics of the study population are summarised in Table 1. The mean age of the cohort was 53.28±14.74 years, with 53.6% being male. The mean serum uric acid level was 6.14±1.64mg/dl. Table 2 presents the mean levels of some cardiovascular risk factors stratified by gender. Serum uric acid levels were higher in men compared with women (6.19±2.32 vs 5.03±2.11mg/dl; p<0.01), while total cholesterol was higher in women (206.40±45.10 vs 188.29±40.23mg/dl; p=0.01).

**Table 1:** Sociodemographic, laboratory and anthropometric characteristics of the patient cohort HDL: high-density lipoprotein.

Factors	Frequency	Percentage
Age in years		
30-44	37	26.8
45-64	72	52.2
65-74	29	21.0
Gender		
Female	64	46.4
Male	74	53.6
Smoking		
Yes	8	5.8
Hypertension		
Yes	105	76.1
Atherogenic Index of Plasma		
High risk	114	82.6
Intermediate risk	19	13.8
Low risk	5	3.6
Triglyceride (mg/dl)		
Elevated	47	34.1
Normal	91	65.9
Uric acid level (mg/dl)		
Elevated	61	44.2
Normal	77	55.8
Framingham Risk Score		
High	45	32.6
Medium	29	21.0
Low	64	46.4
HDL cholesterol (mg/dl)		
Normal	106	76.8
Reduced	32	23.2
Total cholesterol (mg/dl)		
Elevated	65	47.1
Normal	73	52.9
Obesity (kg/m^2^)		
Yes	73	52.9
Waist circumference (cm)		
Central obesity present	119	86.2
Diabetes		
No	112	81.2
Yes	26	18.8

**Table 2:** Gender classification of the means and standard deviations of CVD risk factors and serum uric acid within the study population p<0.05 considered significant. CVD: cardiovascular disease, HDL: high-density lipoprotein.

Risk Factors	Unit	Men (n=74)		Women (n=64)		
		Mean	SD	Mean	SD	p-value
Age	years	53.59	10.01	52.69	13.14	0.65
Systolic blood pressure	mmHg	135.47	20.04	138.02	24.85	0.51
Diastolic blood pressure	mmHg	79.18	13.10	80.00	12.52	0.71
Waist circumference	cm	104.61	13.29	102.05	14.32	0.28
Body mass index	kg/m^2^	31.30	6.43	31.74	6.66	0.69
Total cholesterol	mg/dl	188.29	40.23	206.40	45.10	0.01
HDL cholesterol	mg/dl	52.98	11.33	53.39	11.79	0.84
Uric acid	mg/dl	6.19	2.32	5.03	2.11	<0.01
Triglyceride	mg/dl	152.05	61.92	137.45	45.87	0.12

Table 3 shows the prevalence of hyperuricemia amongst cardiovascular risk factors. Hyperuricemia was more prevalent among individuals with central obesity than those without (93.4 vs 6.6%; p=0.03). The coefficients of correlation between serum uric acid, the FRS, AIP and other cardiovascular risk factors are shown in Table 4. Serum uric acid had a significant positive correlation with FRS (coefficient of correlation: 0.190, p<0.05) and AIP and triglyceride levels (coefficients of correlation: 0.294 and 0.259, respectively; p<0.001). This correlation remained present and significant in multivariate analysis even after adjustment for various cardiovascular risk factors (Tables 5, 6, p<0.001 in all models).

**Table 3:** Hyperuricemia prevalence in CVD risk factors p<0.05 considered significant. CVD: cardiovascular disease, HDL: high-density lipoprotein.

Risk Factor Categories	Hyperuricemia (%)	p-Value
Gender		
Male	52.5	0.81
Female	47.5	
Smoking		0.01
No	88.5	
Yes	11.5	
Diabetes		0.83
No	82.0	
Yes	18.0	
Waist circumference		0.03
Central obesity absent	6.6	
Central obesity present	93.4	
Total cholesterol		0.45
Elevated	50.8	
Normal	49.2	
Body mass index		0.25
Normal	11.5	
Obese	60.7	
Overweight	27.9	
HDL cholesterol		0.95
Normal	77.0	
Reduced	23.0	
Framingham Risk Score		0.26
High	34.4	
Low	39.3	
Medium	26.2	
Triglycerides		0.94
Elevated	34.4	
Normal	65.6	
Atherogenic Index of Plasma		0.85
Increased risk	96.7	
Low/intermediate risk	3.3	
Hypertension		0.81
No	41.0	
Yes	59.0	

**Table 4:** Correlation of serum uric acid levels with CVD risk factors, AIP and FRS *p<0.05 thus significant. CVD: cardiovascular disease, AIP: Atherogenic Index of Plasma, FRS: Framingham 10-year Cardiovascular Risk Score, HDL: high-density lipoprotein.

Variables	Uric Acid	Framingham Risk Score	Atherogenic Index of Plasma
Uric acid	1	0.190*	0.294*
Age	0.106	0.626**	0.020
Framingham Risk Score	0.190*	1	0.081
HDL cholesterol	-0.132	-0.049	-0.614*
Total cholesterol	-0.028	-0.008	-0.213*
Triglycerides	0.259**	0.044	0.823*
Atherogenic Index of Plasma	0.294**	0.081	1
Systolic blood pressure	0.140	0.499*	0.041
Diastolic blood pressure	0.102	0.039	-0.004
Waist circumference	0.110	0.117	0.215*
Body mass index	0.018	0.001	0.111
Smoking	-0.009	-0.021	-0.109
Diabetes	0.078	0.586**	0.041

**Table 5:** Associations of the Framingham Risk Score with the uric acid level in multivariate analyses Model 1: Adjusted for age, systolic and diastolic blood pressure and high-density lipoprotein (HDL) cholesterol. Model 2: Adjusted for age, systolic and diastolic blood pressure, HDL cholesterol, body mass index and triglyceride. Model 3: Adjusted for age, systolic and diastolic blood pressure, HDL cholesterol, body mass index, triglyceride and total cholesterol. Model 4: Adjusted for age, systolic and diastolic blood pressure, HDL cholesterol, body mass index, triglyceride, total cholesterol, diabetes history, waist circumference and smoking history. p<0.05 considered significant. FRS: Framingham 10-year Cardiovascular Risk Score.

Dependent Variable FRS	Standardised Coefficient β	p-Value
Model 1	0.061	<0.001
Model 2	0.057	<0.001
Model 3	0.055	<0.001
Model 4	0.024	<0.001

**Table 6:** Associations of the AIP with the uric acid level in multivariate analyses Model 1: Adjusted for age, systolic and diastolic blood pressure and high-density lipoprotein (HDL) cholesterol. Model 2: Adjusted for age, systolic and diastolic blood pressure, HDL cholesterol, body mass index and triglyceride. Model 3: Adjusted for age, systolic and diastolic blood pressure, HDL cholesterol, body mass index, triglyceride and total cholesterol. Model 4: Adjusted for age, systolic and diastolic blood pressure, HDL cholesterol, body mass index, triglyceride, total cholesterol, diabetes history, waist circumference and smoking history. p<0.05 considered significant. AIP: Atherogenic Index of Plasma.

Dependent Variable AIP	Standardised Coefficient β	p-Value
Model 1	0.211	<0.001
Model 2	0.023	<0.001
Model 3	0.023	<0.001
Model 4	0.021	<0.001

## Discussion

The study’s main aims were to report the general prevalence of hyperuricemia among urban Nigerians attending a cardiology service and to determine if serum uric acid level was an independent risk factor for cardiovascular disease in the Nigerian population. This paper presents evidence supporting the independent association of hyperuricemia with cardiovascular disease risk even after adjustment for other well-known risk factors such as hypertension and diabetes.

The prevalence of hyperuricemia was 44.2% in our study population, much higher than the 2.3%, 17.2% and 25% reported by previous studies, respectively, in Nigeria [15-17]. The prevalence in males was 52.5%, also higher than the 35.8% reported by Ewenighi et al. [18]. This difference could be explained by the fact that the majority of the patients who present at the hospital have cardiovascular risk factors like hypertension and obesity, which are associated with increased oxidative stress, thus leading to elevated levels of sUA [2]. The mean sUA level in males was also significantly higher than in females (6.19±2.32 vs 5.03±2.11mg/dl; p<0.01). This result is comparable with that obtained by Ioachimescu and colleagues (6.5±1.5 vs 5.4±1.7mg/dl) [6] and supports a published study by Alikor et al. showing a correlation between sUA and gender [16].

Fasae et al. showed a strong positive correlation between serum uric acid and central obesity (as measured by waist circumference), an anthropometric marker of cardiovascular risk [19]. Our study found that elevated uric acid levels were significantly more common among patients with central obesity, with a positive correlation, although not significant.

This paper also reports the significant positive correlation between sUA, FRS and AIP. These associations remain significant even after adjustments for various potential confounding risk factors. Our results align with previously published studies in different populations [2,20-24]. The I-Lan Longitudinal Aging Study (ILAS) conducted in I-Lan County, Taiwan, had 973 nonhypertensive and nondiabetic subjects 50 years and older enrolled in a study to find the association between sUA and FRS [21]. This study showed a positive and significant correlation between sUA levels and the Framingham 10-year Cardiovascular Risk Score even after adjustments were made during multivariate analyses for age, BMI, SBP and triglyceride level, amongst other CV risk factors [21]. A similar result was obtained from a similar study, albeit amongst Koreans [20]. As for AIP, Bardak et al. found a positive correlation between sUA and AIP in a cohort study carried out amongst 225 cases and 215 controls in Turkey [2].

High serum uric acid levels have been associated with endothelial dysfunction [25]. Endothelial dysfunction, in turn, is well known for contributing to the pathogenesis of various cardiovascular diseases and risk factors such as diabetes, hypertension, atherosclerosis and obesity [26]. Also, the use of allopurinol in the management of gout has been demonstrated to reduce mortality from heart failure and myocardial infarction as well as death from all causes in patients with hyperuricemia [27].

The study population's high serum uric acid levels reflect an increased CVD risk. Further prospective studies would add to the existing literature by determining if the pharmacological treatment of hyperuricemia can decrease CVD risk scores.

Strengths of this study

Our paper is one of the first out of Nigeria to directly correlate serum uric acid levels to increased cardiovascular risk as measured using the FRS and AIP. Our findings contribute to the global discussion surrounding the relationship between sUA levels and cardiovascular risk and provide evidence unique to the Nigerian population. The study utilised the clinical notes of patients attending a cardiology service and thus was carried out without any invasive measures being applied to patients.

Limitations of this study

This study was a retrospective one. As such, limited conclusions could be drawn from the observed relation between uric acid and cardiovascular risk. A prospective study would be better suited to confirm hyperuricemia's causative effect on cardiovascular disease. This study was also carried out in a hospital catering to cardiovascular conditions. Therefore, the patients presenting here are more likely to have more risk factors for CVD than the general population. This limits the ability of these results to be applied to the general population. We also did not look at current drug therapies and serial uric acid values for the selected patients to try and deduce if a reduction of sUA levels would lead to a reduction of cardiovascular risk.

## Conclusions

The prevalence of hyperuricemia among individuals presenting to the cardiac hospital in Lagos, Nigeria, was high at 52.5% for males and 47.5% for females. Serum uric acid was found to be significantly and independently associated with the FRS and AIP in this urban Nigerian population. Prospective studies in this population group need to determine if this association is causal and if pharmacological reduction of uric acid levels could provide cardiovascular benefit.
